# Microgravity and Cardiovascular Health in Astronauts: A Narrative Review

**DOI:** 10.1002/hsr2.70316

**Published:** 2025-01-07

**Authors:** John Azariah, Umberto Terranova

**Affiliations:** ^1^ Faculty of Medicine and Health Science Crewe Campus, University of Buckingham Crewe UK

**Keywords:** astronauts, cardiovascular adaptations, cardiovascular health, microgravity, spaceflight

## Abstract

**Background:**

Space exploration has become a major interest for scientific and medical research. With increasing duration and frequency of manned space missions, it is crucial to understand the impact of microgravity on the cardiovascular health of astronauts. We focus on this relationship by reviewing literature that explores how microgravity affects several hemodynamic parameters and cardiovascular biomarkers.

**Methods:**

We conducted a search updated to November 2024 across several databases, including PubMed, Cochrane Library, ESA, NASA and DLR, using relevant MeSH terms and selection criteria.

**Results:**

The 22 selected articles detail how microgravity impacts the cardiovascular system and its adaptations. We identify some clear patterns, such as loss of ventricular mass and increased QT intervals (corrected for heart rate) indicating increased risk of arrhythmias. Our analysis confirms that head‐down tilt is an accurate analog of microgravity.

**Conclusions:**

While a direct link between microgravity and cardiovascular disease, such as coronary heart disease and myocardial infarction, remains elusive, the documented physiological changes pose a potential threat to the astronauts' health. We suggest that future research focus on long‐term effects, particularly on female subjects.

## 1 Introduction

Space exploration represents the pinnacle of human ingenuity and curiosity, driving advancements in various fields, including medicine. As investments pour into extended manned missions, it becomes crucial to understand the effects of spaceflight on astronauts.

The space environment contains a number of risk factors for cardiovascular disease (CVD). In deep space, astronauts are inevitably exposed to heavy ions that damage the vascular endothelium [1]. Cosmic radiation might be responsible for the higher CVD mortality rate observed among Apollo lunar astronauts than low Earth orbit and non‐flight astronauts [2]. Disruption of circadian rhythms due to the absence of a natural day‐night cycle may also alter cardiovascular function [3]. For example, data from the Mars500 project, obtained during a 520‐day isolation experiment, revealed a progressive decrease in the heart rate (HR) and a progressive increase in the amplitude of very low and high frequency components of HR variability [4]. Recent findings have revealed similarities between the changes in metabolites released by the gut microbiota during spaceflight and those associated with CVD, suggesting that modulating the gut microbiota could be a promising strategy to improve cardiovascular health in astronauts [5, 6].

Microgravity, characterized by a gravitational force reduced to 1/1,000,000 of the Earth's gravitational force, creates an environment in which individuals appear “weightless“ [7]. This unique condition manifests when objects are at substantial distances from Earth or when they are in free‐fall within Earth's orbit, as exemplified by satellites and the International Space Station.

The human body, including cardiovascular physiology, is adapted to function optimally under gravity. It is therefore not surprising that exposure to microgravity poses high risks to the cardiovascular system, as evidenced by the enhanced coagulation state in the cephalad venous system [8]. However, this topic is controversial. A secondary analysis of data from a NASA longitudinal study, for example, concluded that astronauts and non‐astronauts have a similar risk of CVD [9].

In this review, we analyse the link between microgravity and cardiovascular health, which is essential to ensure the safety of astronauts during spaceflight and upon return to Earth. Research on this area has traditionally focussed on hemodynamic parameters, as changes in HR, cardiac output and blood pressure are among the most immediate effects of microgravity [10, 11]. Incorporating a variety of previously unreported cardiovascular biomarkers and parameters—including microvolt T‐wave alternans (MTWA), stimulating growth factor 2 (ST2) and serum electrolyte levels—alongside traditional hemodynamic measures, we provide a comprehensive examination of the subject. Our findings can inform the development of countermeasures on extended space missions, but more research remains necessary to ensure the safety and well‐being of astronauts.

This study was originally conceived as a systematic review, but due to the considerable heterogeneity in the measured cardiovascular parameters, we chose to present it as a narrative review. This format is more suitable for the qualitative synthesis of the varied results. The rest of this narrative review is structured as follows: First, we outline the methods used to select the studies; second, we report the results of our qualitative synthesis; third, we discuss the significance of these results; finally, we draw conclusions.

## 2 Methods

A literature search was conducted using various databases, including PubMed, Cochrane Library, ESA, NASA and DLR. The search strategy involved using MeSH terms, such as “Cardiovascular Disease” and “Weightlessness,” along with other keywords.

Studies with species other than human, as well as those with populations having pre‐existing cardiovascular medical conditions and risk factors for CVD, were excluded. However, it is important to note that animal models of microgravity have provided valuable insights into cardiovascular changes relevant to humans [12]. Head‐down tilt (HDT), a method where subjects are placed supine on a bed that is tilted (generally by 6°) to elevate the feet, exhibits the strongest comparability to microgravity in spaceflight [13]. Accordingly, we have also included HDT studies in this review, excluding other analogs instead. Additionally, parabolic flight, although an excellent simulation of microgravity in space, was not considered due to its short‐duration of exposure (25 s). Only peer‐reviewed studies published in English and focussing on the relationship between microgravity and CVD were included. The review includes individuals with age in the interval 19–54, which mirrors a naturally lower risk of cardiovascular disease [14] and better represents astronauts. Including individuals who are 20 years older than the typical astronaut age of 34 enables the observation of the long‐term effects of exposure to microgravity. Only studies published after the year 2000 were included in this review, as significant advancements in space exploration have taken place in the last two decades. Studies included are assumed to be informed by relevant research of the past.

## 3 Results

**Figure 1:**
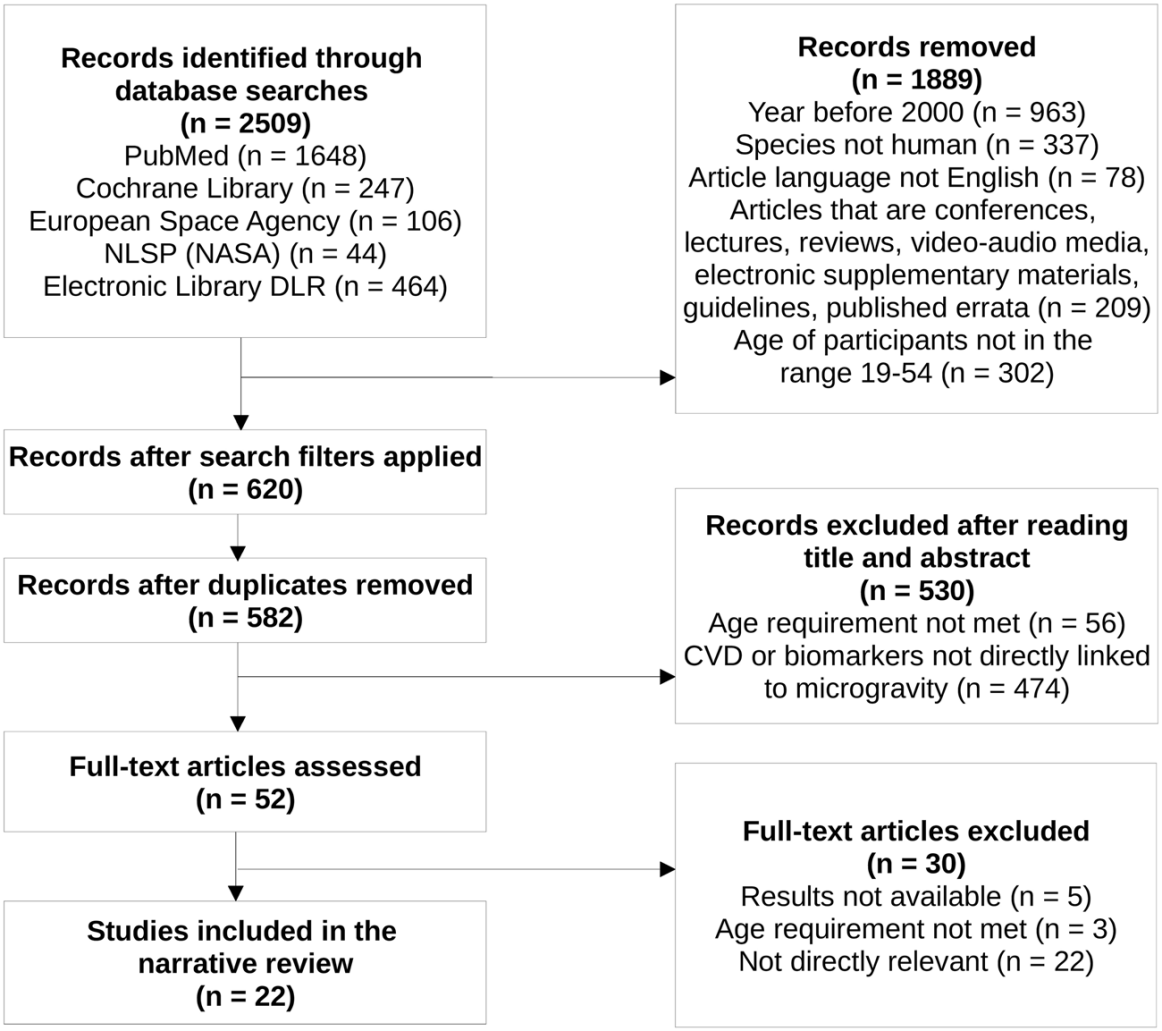
Diagram for the selection of the 22 studies of this review.

Figure 1 shows the different stages of the article selection process. Initially, 2509 studies were identified. After applying the selection criteria, 1889 studies were excluded. Following the removal of 38 duplicates, 582 articles were screened, resulting in the exclusion of 529 studies. Most of them were excluded because relevant search terms appeared in the title or abstract, but they were not directly linked to microgravity. After screening the full texts of 52 articles, five were excluded due to undisclosed results or lack of full publication. Three studies had subjects outside the age range of 19–54 years, while 22 were excluded after a full‐text examination revealed that they were not directly relevant. As a result, 22 peer‐reviewed studies were selected for this review. Of these, 20 were before‐after and two were high‐quality case‐control studies. The 22 publications presented significant clinical heterogeneity, focussing on different aspects of cardiovascular health, such as blood pressure, heart rate and cardiac output.

In the following, we define short‐ and long‐term an exposure to microgravity lasting less or more than 30 days, respectively [15]. In addition to the duration of exposure, we have grouped the results according to the type of exposure to microgravity (HDT or spaceflight).

### 3.1 HDT Effects on the Cardiovascular System

We report below the HDT effects on the cardiovascular system following both short‐ and long‐term exposure to microgravity. These have also been summarized in Table 1.

**Table 1:** Head‐down tilt effects on the cardiovascular system.

Parameter	Change	Length of exposure	Reference	
Stroke volume	15% decrease	Short‐term	Capelli et al. [16]	
	2.3% decrease	Short‐term	Westby et al. [17]	
	Qualitative decrease	Short‐term	Trappe et al. [18]	
	None	Long‐term	Rabineau et al. [19]	
Resting blood pressure	16.7% increase	Long‐term	Fortrat et al. [20]	
Cardiac output	8.7% decrease	Short‐term	Westby et al. [17]	
	Qualitative decrease during exercise	Short‐term	Capelli et al. [16]	
	Slight qualitative decrease	Long‐term	Rabineau et al. [19]	
Arterial stiffness	23% CAVI increase	Long‐term	Rabineau et al. [19]	
Orthostatic tolerance	78.5% decrease in OT test time	Short‐term	Martín‐Yebra et al. [25]	
	57% decrease in number of participants able to complete stand test	Long‐term	Fortrat et al. [20]	
Microvolt T‐wave alternans	25% increase	Short‐term	Grenon et al. [27]	
Serum electrolytes (Na^+^, Cl^‐^, K^+^, Mg^+^^+^)	0.3 mmol/L decrease for K^+^	Long‐term	Sakowski et al. [21]	
O_2_ pulse	12% decrease during submaximal exercise	Short‐term	Trappe et al. [18]	
	13% decrease during maximal exercise	Short‐term	Trappe et al. [18]	
QTc interval	2.9 ms increase	Long‐term	Sakowski et al. [21]	
Ventricular mass	13 g LV + 10 g RV loss	Long‐term	Dorfman et al. [22]	
	20.3 g LV loss	Long‐term	Westby et al. [17]	
Heart rate	18% increase	Short‐term	Capelli et al. [16]	
	11% increase	Short‐term	Trappe et al. [18]	
	20.2% increase	Long‐term	Fortrat et al. [20]	
	54% decrease during sustained handgrip	Long‐term	Spaak et al. [23]	
Mean arterial pressure	7.3 mmHg increase at rest	Long‐term	Spaak et al. [23]	
	43% decrease during sustained handgrip	Long‐term	Spaak et al. [23]	
ST2 and NT‐proBNP levels	Decrease with normalization after recovery	Short‐term	Popova et al. [24]	
D‐dimer levels	None	Short‐term	Popova et al. [24]	

#### 3.1.1 Short‐Term Exposure

During exposure to microgravity, an increase in HR has been observed. Specifically, Capelli et al. [16] reported an average increase of 18% in resting HR following a 14‐day short‐term exposure. In a similar context, Trappe et al. [18] documented a comparatively less pronounced increase of 11% in HR over a period of 17 days of HDT.

Capelli et al. [16] observed a decrease of 15% in stroke volume (SV) over a 14‐day period. Conversely, Westby et al. [17] reported a smaller decrease of 2.3% in SV after 21 days, despite similar study designs and sample sizes. Trappe et al. [18] did not provide specific SV values but indicated a decrease over a 17‐day period in a sample of 8 subjects.

In relation to cardiac output, Westby et al. [17] reported an 8.7% decrease on the 7th day of HDT. Conversely, Capelli et al. [16] found no significant change in cardiac output during rest, but noted a slight decrease when subjects were engaged in exercise.

Orthostatic intolerance is prevalent among cosmonauts. Martín‐Yebra et al. [25] demonstrated a 78.5% reduction in the orthostatic tolerance (OT) test time following a 5‐day exposure to HDT. Xiao et al. [26] suggested that autonomic impairment may reduce OT, but their use of a 4° HDT may limit direct comparability with other studies.

In a study by Grenon et al. [27], 24 men were subjected to short‐term HDT exposure ranging from 9 to 16 days, resulting in a 25% increase in positive MTWA outcomes. However, it is important to note that the study employed a higher threshold for positivity compared to global clinical thresholds. This suggests that positive results may indicate a higher risk of developing arrhythmias as a result of the exposure. Among the four subjects who showed positive results during exposure, two showed negative results after exposure ended. However, five subjects received the drug “midodrine” as part of another study. While no significant impact was expected by the authors, it would have been preferable to exclude those subjects from the analysis.

Trappe et al. [18] conducted a study to assess the effects of HDT on 8 male participants over a period of 17 days, focusing on both maximal and submaximal exercise. The findings revealed a 12% decrease in O_2_ pulse (measured in ml O_2_ per beat) during submaximal exercise and a 13% decrease during maximal exercise by Day 13 of exposure. Additionally, O_2_ consumption showed a 10% decrease by Day 13 of exposure during maximal exercise.

Popova et al. [24] used two biomarkers of myocardial stress, ST2 and N‐terminal prohormone of brain natriuretic peptide (NT‐proBNP) to assess cardiovascular damage in 12 male subjects. Both biomarkers decreased over the 21‐day experiment but returned to baseline levels during recovery. No significant change was observed in the blood levels of d‐dimer, a biomarker associated with thrombosis. Kashirina et al. [28] studied the composition of blood proteins related to the cardiovascular system, identifying all proteins whose concentrations changed after 21 days of exposure to HDT. The authors reported the upregulation of 17 proteins involved in functions, such as the complement and coagulation cascades, acute phase response, platelet degranulation and blood coagulation. In contrast, 8 downregulated proteins were involved in functions ranging from mRNA splicing to cell cytoskeleton remodeling and oxygen transport.

#### 3.1.2 Long‐Term Exposure

In the context of long‐term exposure, Rabineau et al. [19] found that SV remained stable from Day 21 to Day 56 of HDT.

A significant reduction in left ventricle (LV) mass of 13‐grams after 60 days of uninterrupted HDT was noted by Dorfman et al. [22], compared to a 20.3‐gram loss noted by Westby et al. [17].

Westby et al. [17] also observed that after 6 days of recovery, the heart regained 93.3% of its original mass. Additionally, Dorfman et al. [22] reported a 10‐gram loss in right ventricle mass.

Fortrat et al. [20] documented a 57% decrease in the number of participants able to complete a 30‐min OT test. HR increased by 20.2% and resting blood pressure (BP) was 16.7% higher than normal at rest.

Rabineau et al. [19] observed a slight decrease in cardiac output from HDT day 13 to HDT day 60. The noninvasive arterial stiffness assessment using the cardio‐ankle vascular index (CAVI) increased by 23% by day 56 of HDT, but it returned to its initial value following only 4 days of recovery.

Sakowski et al. [21] measured an increase in the QTc interval (QT interval corrected for heart rate) from 411.8 to 414.7 ms after 90‐day exposure, indicating an elevated risk of ventricular arrhythmias. This study also reported a not clinically significant 7% decrease (0.3 mmol/L) in serum potassium levels, indicating a correlation between changes in QTc interval and serum potassium.

Cardiovascular function was assessed by measuring HR and mean arterial pressure (MAP) during a sustained handgrip (SHG) exercise [23]. During a 120‐day HDT bed rest, resting HR showed no significant changes, but during SHG it decreased by 54% by the end of the bed rest period. MAP during SHG decreased by 43% on day 0 after bed rest, while at rest there was a 7.5 mmHg increase on Day 0 post‐experiment. However, the study lacked information on the gender of the subjects, which could have provided additional context to the findings.

### 3.2 Spaceflight Effects on the Cardiovascular System

We report below the spaceflight effects on the cardiovascular system following both short‐ and long‐term exposure to microgravity. These have also been summarized in Table 2.

**Table 2:** Spaceflight effects on the cardiovascular system.

Parameter	Change	Length of exposure	Reference	
O_2_ pulse	10% decrease during maximal exercise (recovery Day 4)	Short‐term	Trappe et al. [18]	
	11% decrease during maximal exercise (recovery Day 13)	Short‐term	Trappe et al. [18]	
Stroke volume	29% decrease	Short‐term	Perhonen et al. [29]	
	Qualitative decrease while standing (no change while supine)	Short‐term	Blomqvist et al. [30]	
Heart rate	14% increase postflight (supine)	Short‐term	Migeotte et al. [31]	
	Average 21 bpm increase postflight (standing)	Long‐term	Baevsky et al. [32]	
	Initial decrease of 26% then 15% increase (standing)	Short‐term	Migeotte et al. [31]	
	3.6% decrease in‐flight then 19.4% increase postflight	Short‐term	Liu et al. [33]	
	19 bpm decrease at rest and qualitative decrease during sustained handgrip	Short‐term	Spaak et al. [23]	
	25% increase in 3/18 participants postflight	Long‐term	Tank et al. [34]	
O_2_ consumption	10.4% decrease (Day 4) and 6% decrease (Day 13)	Short‐term	Trappe et al. [18]	
Cardiac output	19% decrease	Short‐term	Perhonen et al. [29]	
Orthostatic tolerance	64% decrease in participants who completed the stand test	Short‐term	Blomqvist et al. [30]	
	17% of participants had mild‐moderate symptoms during postflight test	Long‐term	Tank et al. [34]	
QTc/R‐R/P‐R interval	Increased QTc in 55% of participants and decreased in rest (not statistically significant)	Short‐term	Mitchell and Meck [35]	
	No change in QTc	Short‐term	D'Aunno et al. [37]	
	Decrease in R‐R	Short‐term	Mitchell and Meck [35]	
	Qualitative decrease in R‐R and P‐R	Short‐term	D'Aunno et al. [37]	
	Decrease in R‐R on Days 1 and 14	Long‐term	Cooke et al. [36]	
	No change in R‐R on landing day	Long‐term	D'Aunno et al. [37]	
	Increase in QTc on landing day and three days after	Long‐term	D'Aunno et al. [37]	
	Increase in P‐R on landing day with a decrease three days after	Long‐term	D'Aunno et al. [37]	
Mean arterial pressure	No change in space at rest	Long‐term	Spaak et al. [23]	
	79% decrease postflight	Long‐term	Spaak et al. [23]	
Blood pressure	Qualitative decrease in PWTT in space with further decrease postflight	Long‐term	Baevsky et al. [32]	
Serum electrolytes (K^+^, Mg^+^^+^, Ca^+^^+^)	No change three days after landing with a 0.4 mmol/L decrease for K+ on landing day	Long‐term	D'Aunno et al. [37]	
Serum electrolytes (K^+^, Mg^+^^+^, Ca^+^^+^)	No change	Short ‐term	D'Aunno et al. [37]	
Ventricular mass	12% decrease in LV	Short‐term	Perhonen et al. [29]	

#### 3.2.1 Short‐Term Exposure

Trappe et al. [18] included four male subjects who experienced microgravity during spaceflight in their study. However, maximal results for Day 13 of spaceflight were not included, unlike the submaximal responses that were assessed. During maximal exercise, the O_2_ pulse decreased by 10% from the initial value by recovery Day 4, and O_2_ consumption decreased by 10.4% within the same timeframe. Significant submaximal responses were observed on Day 13 of spaceflight, with an over 11% decrease in O_2_ pulse and an approximately 6% decrease in O_2_ consumption.

An investigation into cardiac atrophy by Perhonen et al. [29] found a 19% decrease in CO, a 29% decrease in SV and a tendency towards reduced LV mass. This study also included measurements from a supine bed rest group and a control group, providing clear explanations of the results, methods and calculations. Blomqvist et al. [30] examined SV in supine and standing positions and found a significant decrease in SV among the 14 subjects while standing, while SV remained virtually unchanged in the supine position with exposure to microgravity.

Migeotte, Prisk and Paiva [31] conducted a 16‐day spaceflight study on four male subjects, noting a 14% increase in supine HR postflight. The standing HR initially decreased by 26% during flight but increased to 15% above the baseline towards the end of exposure. A similar trend was observed by Liu et al. [33], with HR values decreasing by approximately 3.6% in‐flight but then increasing to 19.4% above the baseline postflight. Spaak et al. [23] found that HR decreased from 76 bpm at rest to 57 bpm on Days 5–6 of spaceflight, with a decrease also observed during SHG.

Blomqvist et al. [30] examined 11 male and 3 female subjects and, although quantitative data was not disclosed, qualitative data indicated a 64% reduction in the number of subjects able to complete a 10‐min stand test. The study concluded that subjects who failed the test exhibited a poorer vasoconstrictor response.

Among the 11 male cosmonauts studied by Mitchell and Meck [35], six exhibited increased QTc intervals, while the remaining five had decreased intervals. However, the variations observed were not clinically significant, with the largest change being 20 ms. In addition, there were no statistically significant changes in the mean QTc intervals. R‐R intervals were found to decrease after flight. D'Aunno et al. [37] also observed a decrease in R‐R and P‐R intervals on landing day following short‐duration spaceflight but did not find prolonged QTc intervals. Notably, the study included seven astronauts who had experienced long‐duration spaceflight, with available electrocardiogram (ECG) data from five of them for comparison purposes.

#### 3.2.2 Long‐Term Exposure

Spaak et al. [23] conducted a study on the effects of four spaceflights of varying durations on the cardiovascular system in four subjects. Resting MAP did not show significant changes in space, but during SHG it decreased in one subject and increased in another, both experiencing significantly reduced values on landing day by 79%.

Eight male cosmonauts during spaceflights lasting 162–196 days were studied and showed a general trend of decreased BP [32]. Photoplethysmography data and preflight ECG analysis were unfortunately not included, and there were no follow‐ups to assess long‐term cardiovascular effects. Pulse‐wave transit time (PWTT), an indicator of BP, was shorter in space and further decreased after spaceflight, suggesting altered cardiac output distribution. BP changes during orthostatic testing were greater postflight according to Tank et al. [34] Baevsky et al. [32] also reported a decrease in respiratory frequency after 5 months.

Cooke et al. [36] noted high end‐tidal CO_2_ levels at the end of an exhaled breath during all protocols, which remained similar during pre and postflight sessions. However, in‐flight CO_2_ measurements could not be obtained due to equipment malfunction.

Functional adaptation of the cardiovascular system in space was proven with increases in HR along with decreases in BP. Baevsky et al. [32] reported an increase in mean HR change while standing postflight, with an average increase of approximately 21 bpm.

A pulse device to measure HR while subjects were standing was utilized by Tank et al. [34]. Preflight, all astronauts completed the standing test without orthostatic symptoms, except for one astronaut who showed an HR increase of over 30 bpm. Postflight, three out of 18 subjects experienced mild to moderate orthostatic symptoms, including light‐headedness and headache, along with a 25% increase in HR. However, all astronauts were able to complete the test without presyncope or overt syncope at 3–5 days after landing.

Cooke et al. [36] conducted a study on three male cosmonauts over a period of 9 months, but incomplete datasets were obtained due to unrelated medical problems faced by the subjects. Consequently, limited numerical data is available. No consistent HR changes were observed in the study. While microgravity decreased the R‐R interval in all cosmonauts, we stress that only three participants were involved in this study, suggesting caution when comparing these data to those from other studies.

D'Aunno et al. [37] conducted a study for 4 months on six males and one female and found no significant changes in R‐R intervals on the landing day following long‐duration flight. Mean P‐R intervals were instead increased on landing day, while QTc intervals remained elevated 3 days later. Serum electrolyte levels were measured at baseline, upon landing at space stations and postflight, but no significant variations were found in sodium, potassium or calcium 3 days after landing, apart from a clinically insignificant decrease of 0.4 mmol/L in potassium levels on landing day.

## 4 Discussion

Although none of the studies revealed a clear link between microgravity and increased incidence of CVD, the majority of them have demonstrated significant cardiovascular deconditioning. Crucially, we note the extremely small number of female astronauts in the studies reviewed. The lack of existing research makes females even more vulnerable than males to the risks posed by microgravity. In addition, we confirm that HDT is a reliable analog of microgravity in spaceflight, as shown by the fact that both Trappe et al. [18] and Spaak et al. [23] produced similar results for HDT and spaceflight groups.

A commonly known effect of microgravity is the acute fluid shift towards the upper body, often resulting in flu‐like symptoms and increased sympathetic activity with a decrease in central venous pressure. Plasma volume and red cell mass decrease chronically [34]. Vagal baroreflex gain and cardiac neural outflow decrease, the changes occurring early and remaining [36]. The decreased cardiac capacity likely causes a reduced cardiovascular response to isometric muscle action [23]. A decrease in maximal O_2_ uptake is also seen, as reported by Martín‐Yebra et al. [25], causing a reduction in OT.

One of the most important cardiovascular changes in microgravity is the loss of ventricular mass, as observed by Perhonen et al. [29], with a 12% decrease in LV mass over 10 days in space. Cardiac atrophy and ventricular remodeling are likely to occur due to reduced load on the heart, leading to regression of the cardiac muscle [29]. Although symptoms may not manifest during spaceflight, astronauts' postflight health can be significantly affected.

Long‐duration spaceflight is believed to induce structural and functional alterations in the vasculature that resemble the effects of aging observed on Earth [38, 39]. Microgravity increases levels of retinol‐binding protein 4 (RBP‐4), which is linked to altered insulin sensitivity, further suggesting its role in vascular aging [28]. The increase in arterial stiffness [19], an important predictor of cardiovascular risk, is consistent with the elevated biomarkers of oxidative stress observed during 6 months of spaceflight [40]. Similar to the markers of arterial stiffness, those indicating oxidative stress returned to preflight levels after a few days of recovery, suggesting that the human body can adapt to these changes. This reversibility trend was confirmed by Popova et al. [24], who observed a return to baseline for two biomarkers of myocardial stress, namely ST2 and NT‐proBNP. However, the implications of cardiovascular aging for extended space missions (lasting longer than 6 months) remain largely unexplored.

Migeotte et al. [31] noted a decrease in HR during spaceflight and an increase in HR upon return to Earth, attributing both to decreased sympathetic activity and increased vagal tone rather than only orthostatic stress, as is usually assumed. Baevsky et al. [32] found an increase in mean resting HR variability, concluding that this was a result of either decreased sympathetic HR control or increased parasympathetic control. Results of reduced parasympathetic HR control upon a stand test found by Tank et al. [34] concur that remodeling of the central nervous system occurs during spaceflight, resulting in reduced sympathetic responses to baroreceptor input.

Many studies used ECGs to take cardiovascular measurements through the various intervals. Long‐duration exposure to microgravity prolongs QTc intervals [21, 37], and thereby cardiac conduction and re‐polarization, increasing the risk of developing arrhythmias. However, this is not the case with short‐duration exposure [35, 37]. Sakowski et al. [21] concluded that long‐duration HDT decreases stability of action potentials and uniformity of the heartbeat, thereby increasing proneness to ventricular dysrhythmias. Fortrat et al. [20] noted that alterations in R‐R interval and BP variability were not linked, but correlated to orthostatic hypotension. Ventricular arrhythmias were suspected to be caused by alterations to the cardiac re‐polarization processes that could result in abnormalities in T‐wave, such as alternans [27]. This hypothesis has been supported by Ramírez et al. [41]. It is crucial to mention that in the study by Grenon et al. [27] subjects with positive MTWA showed K^+^ ion losses as well, potentially suggestive causation and potassium supplementation as a countermeasure.

## 5 Conclusions

This narrative review suggests that remodeling of the cardiovascular system occurs in microgravity, affecting several parameters such as orthostatic tolerance, ventricular mass and heart rate. However, whether these factors contribute to long‐term cardiovascular disease is still unknown, posing a concern for future space exploration. Thus, it is essential for the health and safety of both male and female astronauts that further sex‐disaggregated primary research takes place, in addition to diagnostic, treatment and prevention strategies.

## Author Contributions

**John Azariah:** writing–original draft, methodology, investigation. **Umberto Terranova:** writing–review and editing, supervision.

## Ethics Statement

This narrative review was not subject to ethical approval as only previously published data was reported.

## Conflicts of Interest

The authors declare no conflict of interest.

## Transparency Statement

The lead author Umberto Terranova affirms that this manuscript is an honest, accurate, and transparent account of the study being reported; that no important aspects of the study have been omitted; and that any discrepancies from the study as planned (and, if relevant, registered) have been explained.

## Supporting information

Supporting information.

## Data Availability

The data that supports the findings of this study are available in the supplementary material of this article.
