# Supplementary material for: Microgravity and Cardiovascular Health in Astronauts: A Narrative Review
Source: Health Sci Rep. 2025 Jan 7;8(1):e70316. doi: 10.1002/hsr2.70316 (PMC11705478; doi:10.1002/hsr2.70316)
Supplement: Supplementary file 1 — Supporting information. [file HSR2-8-e70316-s001.pdf]

Microgravity and Cardiovascular Health in Astronauts: A Narrative Review (Supplementary Information)

John Azariah and Umberto Terranova

Faculty of Medicine and Health Science, Crewe Campus, University of Buckingham, Crewe, CW1 5DU, United Kingdom

Search strategy.

**Main Category**	**Specific Category**	**Keywords in Boolean Search Format**	**Search Number**
Population	Inclusion criteria	‘Human’ OR ‘humans’ OR ‘astronauts’ OR ‘subjects’ OR ‘participants’	1
	Exclusion criteria	Animal’ OR ‘animals’ OR ‘Animalia’ OR ‘plants’ OR ‘plantae’ OR ‘prokaryotes’ OR ‘botany’	2
Boolean		#1 NOT #2	3
Reduced gravity	Microgravity and ground based analogues	Microgravity’ OR ‘Hypogravity’ OR ‘Weightlessness’ OR ‘Reduced gravity’ OR ‘Gravitational unloading’ OR ‘Head-down bed rest’ OR ‘Head-down tilt bed rest’ OR ‘head-down tilt position’ OR ‘head-low tilt position’ OR ‘head-down tilt posture’ OR ‘head-low tilt posture’	4
Responses	Cardiovascular disease	Cardiovascular Diseases/blood’ OR ‘Cardiovascular Diseases/classification’ OR ‘Cardiovascular Diseases/complications’ OR Cardiovascular Diseases/diagnosis’ OR ‘Cardiovascular Diseases/enzymology’ OR ‘Cardiovascular Diseases/epidemiology’ OR ‘Cardiovascular Diseases/aetiology’ OR ‘Cardiovascular Diseases/microbiology’ OR ‘Cardiovascular Diseases/mortality’ OR ‘Cardiovascular	5

		Diseases/pathology' OR 'Cardiovascular Diseases/pathophysiology' OR 'Cardiovascular Disease' OR 'Cardiovascular Diseases' OR 'CVD' OR 'Heart' OR 'Major Adverse Cardiac Events' OR 'Cardiac Events' OR 'Cardiac Event' OR 'Adverse Cardiac Event' OR 'Adverse Cardiac Events' OR 'Heart disease' OR 'Coronary heart disease' OR 'CHD' OR 'Myocardial infarction' OR 'Heart attack' OR 'Atherosclerosis' OR 'Hypertension' OR 'High blood pressure' OR 'Ischemic heart disease' OR 'Arrhythmia' OR 'Cardiomyopathy' OR 'Heart failure' OR 'Valvular heart disease' OR 'Angina' OR 'Aortic aneurysm' OR 'Rheumatic heart disease' OR 'Cardiac arrest' OR 'Sudden cardiac death' OR 'Congestive Heart Failure' OR 'Atrial Fibrillation' OR 'Ventricular Fibrillation' OR 'Bradycardia' OR 'Tachycardia' OR 'Angina pectoris' OR 'Aortic aneurysm' OR 'Pericarditis'	
Responses	Cardiovascular disease biomarkers	Biomarkers for Cardiovascular Disease' OR 'Biomarkers for Cardiovascular Diseases' OR 'cholesterol' OR 'triglycerides' OR 'C-reactive protein' OR 'CRP' OR 'high-sensitivity C-reactive protein' OR 'hs-CRP' OR 'troponin' OR 'creatine kinase' OR 'lactate dehydrogenase' OR 'brain natriuretic peptide' OR 'BNP' OR 'N-terminal pro b-type natriuretic peptide' OR 'NT-proBNP' OR 'Homocysteine' OR 'Myeloperoxidase' OR 'Adiponectin' OR 'Fibrinogen' OR 'Lipoprotein-associated phospholipase A2' OR 'Lp-PLA2' OR 'Matrix metalloproteinases' OR 'MMPs' OR 'Osteoprotegerin' OR 'OPG' OR 'Paraoxonase-1' OR 'PON-1' OR 'Soluble receptor for advanced glycation end products' OR 'sRAGE' OR 'Tumor necrosis factor-alpha' OR 'TNF-alpha' OR 'apolipoprotein B' OR 'apolipoprotein A/apolipoprotein B ratio' OR 'high density lipoprotein' OR 'cardiac troponins' OR 'troponins' OR 'troponin I' OR 'troponin T' OR 'serum creatinine' OR 'cystatin C' OR 'LDL/HDL ratio' OR 'soluble urokinase plasminogen activator receptor' OR 'suPAR' OR 'oxidative biomarkers' OR 'myeloperoxidase' OR 'reactive oxygen species' OR 'ROS's' OR 'endothelial dysfunction biomarkers' OR 'pentraxin-3' OR 'asymmetrical dimethylarginine' OR	6

		‘angiopoietin’ OR ‘high-sensitive troponin’ OR ‘hsTn’ OR ‘gamma-glutamyl transferase’ OR ‘GGT’ OR ‘alkaline phosphatase’ OR ‘AF’ OR 'aspartate transaminase’ OR ‘AST’ OR ‘alanine transaminase’ OR ‘ALT' OR ‘Lipoprotein (a)’ OR ‘Apolipoprotein A-1’ OR ‘Apolipoprotein B’ OR ‘LDL particle size and number’ OR ‘Triglycerides’ OR ‘Cholesterol ester transfer protein’ OR ‘Lipoprotein-associated phospholipase A2’ OR ‘Small-dense LDL’ OR ‘Paraxonase-1’ OR ‘Plasma phospholipid transfer protein’ OR ‘Interleukins 6’ OR ‘Interleukins 10' OR ‘Interleukins 18' OR ‘Tumor necrosis factor alpha’ OR ‘Intercellular adhesion molecule 1’ OR ‘Myeloperoxidase’ OR ‘Vascular cell adhesion molecule’ OR ‘Ferritin’ OR ‘Prothrombotics’ OR ‘D-dimer’ OR ‘Von Willebrand factor’ OR ‘Homocysteine' OR ‘Haptoglobin’ OR ‘Insulin’ OR ‘Adiponectin’ OR ‘Leptin OR Fasting glucose’ OR ‘E-selectin’ OR ‘Chimerin’ OR ‘Cystatin-C’ OR ‘Carotid intima-media thickness’ OR ‘Coronary calcium score’	
Boolean		#5 OR #6	7
Boolean		#3 AND #4 AND #7	8

Quality appraisal of the before-after studies.

**Studies**	**1**	**2**	**3**	**4**	**5**	**6**	**7**	**8**	**9**	**10**	**11**	**Avg**
Baevsky et al., 2007	Red	Green	Green	Green	Yellow	Green	Yellow	Yellow	Green	Green	Red	Yellow
Blomqvist et al., n.d.	Yellow	Green	Green	Green	Green	Green	Yellow	Green	Green	Green	Green	Green
Capelli et al., 2008	Green	Yellow	Green	Green	Yellow	Green	Green	Yellow	Yellow	Green	Green	Green
Cooke et al., 2000	Red	Yellow	Green	Green	Red	Yellow	Green	Green	Yellow	Green	Red	Yellow
D’Aunno et al., 2003	Red	Green	Green	Green	Yellow	Green	Green	Yellow	Green	Yellow	Yellow	Yellow
Dorfman et al., 2007	Green	Green	Green	Green	Yellow	Green	Green	Green	Green	Green	Green	Green
Fortrat et al., 2001	Green	Green	Green	Green	Yellow	Green	Green	Red	Green	Green	Yellow	Green
Grenon et al., 2005	Green	Green	Green	Green	Yellow	Green	Green	Green	Green	Green	Yellow	Green
Kashirina et al., 2024	Green	Green	Green	Green	Red	Green	Green	Yellow	Green	Green	Red	Green
Liu et al., 2015	Yellow	Green	Green	Green	Red	Green	Green	Red	Green	Green	Green	Green
Martin-Yebra et al., 2015	Green	Green	Green	Green	Green	Green	Green	Yellow	Yellow	Green	Green	Green
Migeotte et al., 2003	Yellow	Green	Green	Green	Red	Green	Green	Yellow	Green	Green	Green	Green
Mitchell and Meck, 2004	Red	Green	Green	Green	Yellow	Yellow	Yellow	Green	Yellow	Green	Yellow	Yellow
Popova et al., 2024	Green	Green	Green	Green	Yellow	Green	Green	Yellow	Green	Green	Green	Green
Sakowski et al., 2011	Green	Green	Green	Green	Green	Green	Green	Green	Green	Green	Yellow	Green
Spaak et al., 2001	Red	Yellow	Green	Green	Yellow	Yellow	Green	Green	Yellow	Green	Green	Yellow
Tank et al., 2011	Red	Green	Green	Green	Yellow	Yellow	Yellow	Green	Green	Green	Yellow	Yellow
Trappe et al., 2006	Green	Green	Green	Green	Yellow	Yellow	Green	Green	Green	Green	Green	Green
Westby et al., 2016	Green	Green	Green	Green	Yellow	Green	Green	Yellow	Green	Green	Yellow	Green
Xiao et al., 2004	Green	Green	Green	Green	Yellow	Green	Green	Yellow	Green	Green	Green	Green

Each question below (see https://www.nhlbi.nih.gov/health-topics/study-quality-assessment-tools) received a score (0-red, 1-yellow, or 2-green) reflecting its objective fulfilment.

1. Were eligibility/selection criteria for the study population prespecified and clearly described?

2. Was the study question or objective clearly stated?

3. Were the participants in the study representative of those who would be eligible for the test/service/intervention in the general or clinical population of interest?

4. Were all eligible participants that met the prespecified entry criteria enrolled?

5. Was the sample size sufficiently large to provide confidence in the findings?

6. Was the test/service/intervention clearly described and delivered consistently across the study population?

7. Were the outcome measures prespecified, clearly defined, valid, reliable, and assessed consistently across all study participants?

8. Were the people assessing the outcomes blinded to the participants' exposures/interventions?

9. Was the loss to follow-up after baseline 20% or less? Were those lost to follow-up accounted for in the analysis?

10. Did the statistical methods examine changes in outcome measures from before to after the intervention? Were statistical tests done that provided p values for the pre-to-post changes?

11. Were outcome measures of interest taken multiple times before the intervention and multiple times after the intervention (i.e., did they use an interrupted time-series design)?
